# Effects of Polymethoxyflavonoids on Bone Loss Induced by Estrogen Deficiency and by LPS-Dependent Inflammation in Mice

**DOI:** 10.3390/ph11010007

**Published:** 2018-01-20

**Authors:** Shigeru Matsumoto, Tsukasa Tominari, Chiho Matsumoto, Shosei Yoshinouchi, Ryota Ichimaru, Kenta Watanabe, Michiko Hirata, Florian M. W. Grundler, Chisato Miyaura, Masaki Inada

**Affiliations:** 1 Cooperative Major of Advanced Health Science, Tokyo University of Agriculture and Technology, 2-24-16 Nakacho, Koganei, Tokyo 184-8588, Japan; matshi37@yahoo.co.jp (S.M.); miyaura@cc.tuat.ac.jp (C.M.); 2 Department of Biotechnology and Life Science, Tokyo University of Agriculture and Technology, 2-24-16 Nakacho, Koganei, Tokyo 184-8588, Japan; tominari@cc.tuat.ac.jp (T.T.); c-matsu@cc.tuat.ac.jp (C.M.); s170440s@st.go.tuat.ac.jp (S.Y.); s163362x@st.go.tuat.ac.jp (R.I.); hirata@cc.tuat.ac.jp (M.H.); 3 Institute of Global Innovation Research, Tokyo University of Agriculture and Technology, 2-24-16 Nakacho, Koganei, Tokyo 184-8588, Japan; kenta-w@cc.tuat.ac.jp; 4 Institute of Crop Science and Resource Conservation, University of Bonn, Karlrobert-Kreiten-Strasse 13, 53115 Bonn, Germany; grundler@uni-bonn.de

**Keywords:** polymethoxyflavonoid, bone resorption, osteoporosis, lipopolysaccharide, periodontitis

## Abstract

Polymethoxyflavonoids (PMFs) are a family of the natural compounds that mainly compise nobiletin, tangeretin, heptamethoxyflavone (HMF), and tetramethoxyflavone (TMF) in citrus fruits. PMFs have shown various biological functions, including anti-oxidative effects. We previously showed that nobiletin, tangeretin, and HMF all inhibited interleukin (IL)-1-mediated osteoclast differentiation via the inhibition of prostaglandin E2 synthesis. In this study, we created an original mixture of PMFs (nobiletin, tangeretin, HMF, and TMF) and examined whether or not PMFs exhibit co-operative inhibitory effects on osteoclastogenesis and bone resorption. In a coculture of bone marrow cells and osteoblasts, PMFs dose-dependently inhibited IL-1-induced osteoclast differentiation and bone resorption. The optimum concentration of PMFs was lower than that of nobiletin alone in the suppression of osteoclast differentiation, suggesting that the potency of PMFs was stronger than that of nobiletin in vitro. The oral administration of PMFs recovered the femoral bone loss induced by estrogen deficiency in ovariectomized mice. We further tested the effects of PMFs on lipopolysaccharide-induced bone resorption in mouse alveolar bone. In an ex vivo experimental model for periodontitis, PMFs significantly suppressed the bone-resorbing activity in organ cultures of mouse alveolar bone. These results indicate that a mixture of purified nobiletin, tangeretin, HMF, and TMF exhibits a co-operative inhibitory effect for the protection against bone loss in a mouse model of bone disease, suggesting that PMFs may be potential candidates for the prevention of bone resorption diseases, such as osteoporosis and periodontitis.

## 1. Introduction

The balance between osteoclastic bone resorption and osteoblastic bone formation regulates bone remodeling and bone mass. Osteoclasts are primary bone-resorbing cells and are differentiated from monocyte-macrophage lineage cells. The interaction between receptor activator of NF-κB ligand (RANKL) and RANK is essential for osteoclast differentiation [1,2]. RANKL is expressed on the cell surface of osteoblasts in response to bone-resorbing factors, such as lipopolysaccharide (LPS) and interleukin (IL)-1, whereas RANK is expressed on osteoclast precursor cells [3,4]. Prostaglandin (PG) E_2_ is mainly produced by osteoblasts and acts as a potent inducer of inflammatory bone resorption. LPS and IL-1 are known to stimulate PGE_2_ production by osteoblasts via the upregulation of mRNA expression of cyclooxygenase (COX)-2 and membrane-bound PGE synthase (mPGES)-1, and PGE_2_ induces RANKL expression on the osteoblast surface [5,6]. We previously reported that PGE_2_ has a critical role in inflammatory bone resorption, and PGE_2_ was recognized by its receptor EP4 in osteoblasts to express RANKL, resulting in osteoclast differentiation [7,8]. 

Osteoporosis is the most common bone-related disease, and a decrease in the estrogen level results in severe bone loss in postmenopausal women. Ovariectomized (OVX) mice are widely used as an animal model of postmenopausal osteoporosis. OVX mice exhibit severe bone loss due to estrogen deficiency in the femur, and inflammatory cytokines, such as IL-1, may be involved in the bone loss [9]. Periodontitis is an inflammatory bone disease caused by the infection of mixed Gram-negative bacteria, and the progression of periodontitis results in alveolar bone destruction and tooth loss. LPS is an outer membrane component of Gram-negative bacteria and contributes to the pathogenesis of periodontitis via toll-like receptor (TLR) 4 signaling [10]. We previously established a novel mouse model for periodontitis and reported that LPS-induced alveolar bone loss was attenuated in mPGES-1-deficient mice, suggesting that mPGES-1-mediated PGE_2_ synthesis is essential for LPS-mediated bone loss in periodontitis [6]. We recently examined the relationship between the bone loss induced by OVX and local inflammatory bone destruction in a model of periodontitis [11]. The combination of OVX and LPS treatment induced dramatic bone destruction in the alveolar bone of mice [11]. This is consistent with the finding that both bone disorders are processed simultaneously in patients of advanced age [12]. 

Polymethoxyflavonoids (PMFs) are abundantly present in citrus fruits and exhibit a wide range of biological functions. Nobiletin (5,6,7,8,3′,4′-hexamethoxyflavone) and tangeretin (5,6,7,8,4′-pentamethoxyflavone), which possesses six and five methoxy groups, respectively, have been shown to display various activities, such as anti-cancer, anti-inflammation, and anti-obesity effects [13,14,15,16,17,18,19,20]. Previous studies have shown that heptamethoxyflavone (3,5,6,7,8,3′,4′-heptamethoxyflavone, HMF) regulates inflammation and behavior in the central nervous system [21,22]. We previously found that these PMFs exhibited bone-protective activities. Nobiletin suppresses IL-1-induced osteoclast differentiation via the attenuation of IKK-mediated NF-κB activation in vitro and restores the femoral bone loss in OVX mice [23]. The local injection of nobiletin and tangeretin suppressed LPS-induced alveolar bone loss in a mouse model of periodontitis [24]. HMF also suppressed osteoclast differentiation and LPS-induced bone resorption in an ex vivo experimental model of periodontitis [25]. Recent reports have shown that a PMF mixture of nobiletin, tangeretin, tetramethoxyflavone (5,6,7,4′-tetramethoxyflavone, TMF), and HMF, which are extracted from orange peels, protected against ultraviolet-induced skin damage [26,27,28]. These previous findings suggest that components of PMFs derived from citrus fruit exhibit co-operative effects on various biological functions.

The disadvantage of respective PMFs such as nobiletin is the cost associated with purification to 98% purity. In the present study, we have obtained an original mixture of PMFs (a mixture of nobiletin, tangeretin, HMF, and TMF) to solve the disadvantage of high cost, and examined whether or not the PMFs exert inhibitory effects on osteoclastogenesis and inflammatory bone resorption in vitro and on bone loss in OVX mice. The PMFs may be useful for the prevention and treatment of various bone-related diseases.

## 2. Results

### 2.1. Effects of PMFs on IL-1-Induced Osteoclast Differentiation and Bone Resorption

**Figure 1:**
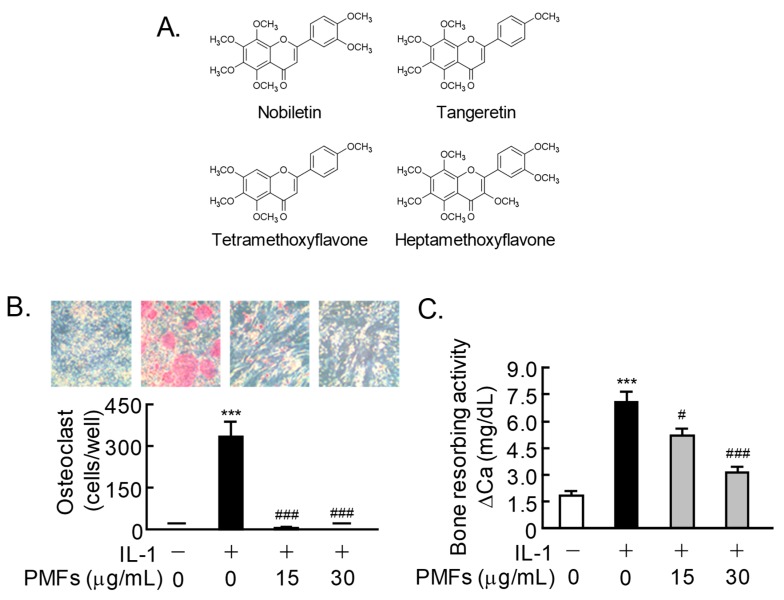
Effects of PMFs on the IL-1-induced osteoclast differentiation and bone resorption. (**A**) Structures of nobiletin, tangeretin, tetramethoxyflavone (TMF), and heptamethoxyflavone (HMF). (**B**) Mouse POBs and BMCs were cocultured for 7 days with IL-1 (2 ng/mL) in the presence of PMFs (15, 30 μg/mL). TRAP-positive multi-nuclear osteoclasts were classified as osteoclasts. The upper panels show TRAP-stained osteoclastic cells. The data are expressed as the means ± SEM (standard error of the mean) of 4 wells. (**C**) Newborn mouse calvariae were cultured for 5 days with IL-1 (4 ng/mL) and PMFs (15, 30 μg/mL). The bone-resorbing activity was determined based on the calcium concentration in the medium using OCPC methods. The data are expressed as the means ± SEM of 5 independent cultures. Significant differences between the two groups are indicated; *** *p* < 0.001 vs. control, ^#^ *p* < 0.05 and ^###^ *p* < 0.001 vs. IL-1.

The PMF mixture used in the present study consisted of nobiletin, tangeretin, TMF, and HMF (35.7%, 11.0%, 2.4%, and 38.8%, respectively). The structures of the four natural compounds are shown in Figure 1A. To examine the effects of PMFs on IL-1-induced osteoclast differentiation, BMCs and POBs were cocultured with or without IL-1 (2 ng/mL) and PMFs (15, 30 μg/mL). The IL-1-induced osteoclast differentiation was completely inhibited by adding PMFs (Figure 1B). In calvarial organ cultures, PMFs (15, 30 μg/mL) dose-dependently suppressed the IL-1-induced bone-resorbing activity (Figure 1C).

### 2.2. Effects of PMFs on Bone Mass in OVX Mice, an In Vivo Model of Osteoporosis

**Figure 2:**
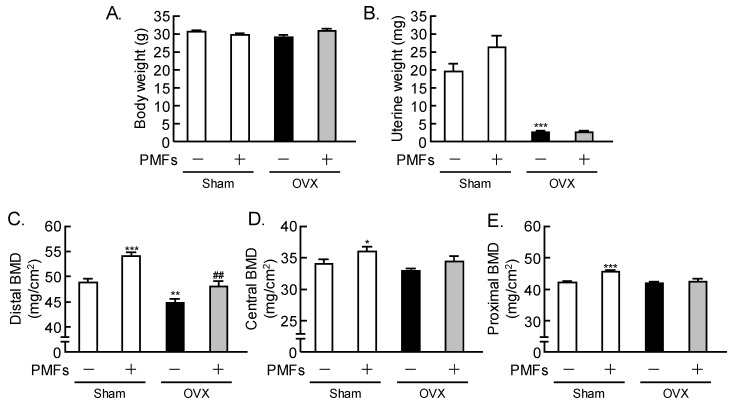
PMFs restored bone loss due to estrogen deficiency in mice. Female mice were ovariectomized (OVX) and sham-operated, and some mice were orally administered PMFs (5 mg/day/mouse) daily. At 4 weeks after the surgery, the body weight (**A**) and uterine weight (**B**) were measured. The distal BMD (**C**), central BMD (**D**), and proximal BMD (**E**) of the femurs were measured by dual X-ray absorptiometry. Data are expressed as the means ± SEM of 6–8 mice. Significant differences between the two groups are indicated; * p < 0.05, ** *p* < 0.01 and *** *p* < 0.001 vs. Sham without PMFs, ^##^ *p* < 0.01 vs. OVX without PMFs.

To determine whether or not PMFs recovered the bone loss due to estrogen depletion in vivo, PMFs were orally administered (5 mg/day/mouse) to sham-operated and OVX mice. OVX induced uterine atrophy due to estrogen loss, and PMFs did not affect the body weight or uterine weight in OVX mice (Figure 2A,B). Estrogen deficiency in OVX mice markedly reduced the femoral BMD (Bone Mineral Density), but the oral administration of PMFs restored the loss of femoral BMD in the distal area (Figure 2C). The BMD in the central area was not reduced by OVX (Figure 2D). In sham mice, the femoral BMD in both the distal and central areas showed a significant increase following the administration of PMFs (Figure 2C,D).

### 2.3. An Analysis of the Femoral Trabecular Bone Using μCT

**Figure 3:**
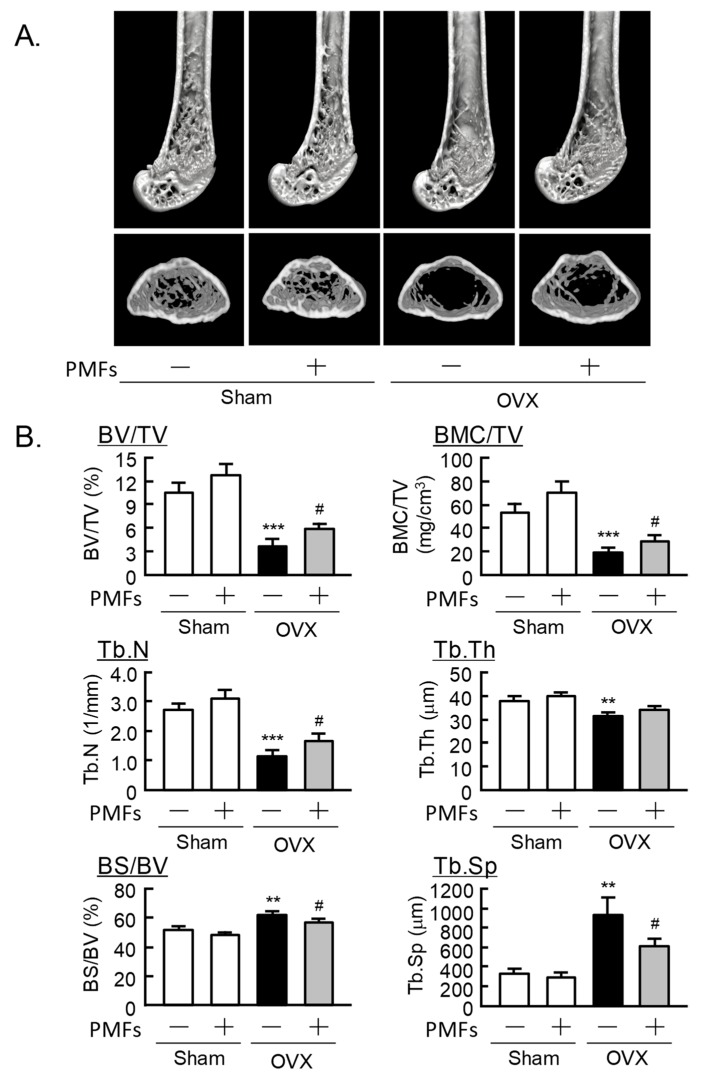
The μCT analysis of the trabecular bone mass of the distal femur in mice. (**A**) Three-dimensional (3D) μCT reconstruction images for the distal femur. (**B**) The parameters (BV/TV, BMC/TV, Tb.N, Tb.Th, BS/BV, and Tb.Sp) of trabecular bone were analyzed using μCT. Data are expressed as the means ± SEM of 6–8 mice. Significant differences between the two groups are indicated; ** *p* < 0.01 and *** *p* < 0.001 vs. Sham without PMFs, ^#^ *p* < 0.05 vs. OVX without PMFs.

We next analyzed the femoral trabecular bone architecture using μCT. Three-dimensional reconstruction images were obtained at the distal femurs (Figure 3A). The parameters of trabecular bone were as follows: bone volume/tissue volume (BV/TV), bone mineral content/tissue volume (BMC/TV), trabecular number (Tb.N) and trabecular thickness (Tb.Th), and bone surface/bone volume (BS/BV) and trabecular separation (Tb.Sp). BV/TV, BMC/TV, Tb.N, and Tb.Th were clearly decreased in OVX mice compared with sham mice, but PMFs significantly restored the loss of BV/TV, BMC/TV, and Tb.N. (Figure 3B). BS/BV and Tb.Sp increased to 119% and 275% in OVX mice compared with sham mice, while the PMFs significantly recovered (Figure 3B). PMFs did not affect these parameters in sham mice.

### 2.4. Effects of PMFs on LPS-Induced Osteoclast Formation and ex vivo Alveolar Bone Resorption

**Figure 4:**
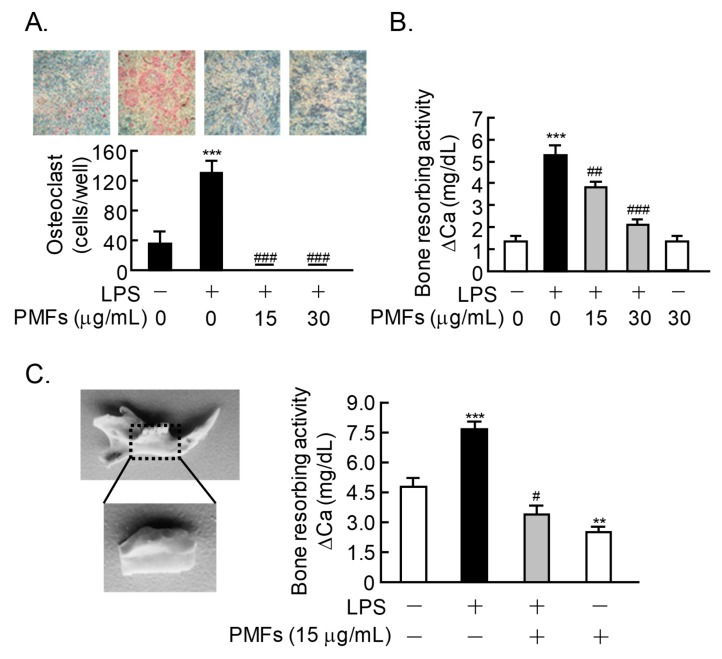
PMFs inhibited LPS-induced osteoclastogenesis and bone resorption in mouse mandibular alveolar bone. (**A**) Mouse POBs and BMCs were cocultured for 7 days with LPS (1 ng/mL) in the presence or absence of PMFs (15, 30 μg/mL). TRAP-positive multi-nuclear osteoclasts were classified as osteoclasts. The upper panels show TRAP-stained osteoclastic cells. The data are expressed as the means ± SEM of 4 wells. (**B**) Newborn mouse calvariae were cultured for 5 days with or without LPS (1 μg/mL) and PMFs (15, 30 μg/mL). (**C**) Mandibular alveolar bone was collected from mouse lower gingiva, and the teeth were removed under a microscope (left panel). Calvariae and alveolar bone were cultured for 5 days with or without LPS (1 μg/mL) and PMFs (15, 30 μg/mL), and the bone-resorbing activity was determined based on the calcium concentration in the medium. Data are expressed as the mean ± SEM of 4 independent cultures. Significant differences between the two groups are indicated; ** *p* < 0.01 and *** *p* < 0.001 vs. control, ^#^ *p* < 0.05, ^##^ *p* < 0.01 and ^###^ *p* < 0.001 vs. LPS.

We reported that LPS initiates inflammatory bone resorption via TLR4 in mice [6]. We established an in vitro model of LPS-induced osteoclastic bone resorption and ex vivo model of alveolar bone resorption to evaluate periodontal bone resorption in mice [24]. In cocultures of BMCs and POBs, LPS induced osteoclast differentiation and bone resorption, and PMFs dose-dependently suppressed this osteoclastogenesis and bone resorption (Figure 4A,B). The effects of PMFs were evaluated using organ culture of alveolar bone, an ex vivo model for periodontitis. Mouse alveolar bone was collected from the mandibular bone by trimming and removing the teeth (Figure 4C, left panel). The LPS-induced bone-resorbing activity in alveolar bone was clearly repressed by adding PMFs (Figure 4C, right panel).

## 3. Discussion

In this study, we used an original PMF mixture consisting of nobiletin, tangeretin, TMF, and HMF (35.7%, 11.0%, 2.4%, and 38.8%, respectively) and showed the protective effects of PMFs against osteoclast differentiation and bone resorption. Yoshizaki et al. [26,28] reported that a PMF mixture derived from orange peel extracts consisting of nobiletin, tangeretin, and HMF (37.3%, 7.9%, and 46.9%) inhibited UVB-induced COX-2 expression via peroxisome proliferator-activated receptor (PPAR) γ activation, and UVB-induced matrix metalloproteinase (MMP)-1 expression via the inhibition of c-jun N-terminal kinase (JNK) phosphorylation in human keratinocyte cell line HaCaT. This group also demonstrated that the PMF mixture inhibited melanogenesis in the human melanoma cell line MH3KO, and that the potency of the PMF mixture was similar to that of nobiletin and HMF, whereas the potency of tangeretin was weaker than that of the other compounds [27]. Another group showed that the number and position of the methoxy groups modulates the anti-tumor activity of PMFs and that nobiletin, tangeretin, and HMF showed equivalent efficacy [29]. We previously reported that nobiletin, tangeretin, and HMF exhibit inhibitory activity against osteoclast differentiation and bone resorption, and that the potency of nobiletin is higher than that of tangeretin and HMF in vitro [23,24,25]. We have reported that bone-resorbing factors such as IL-1 and LPS induce osteoclast differentiation by the induction of RANKL and NFκB in osteoblasts and by the activation of transcription factor NFATc1 in osteoclast precursor cells, and that nobiletin and HMF suppressed the differentiation process into mature osteoclasts [23,24,25]. In addition, nobiletin showed more potent effects for bone protection than tangeretin in a mouse model of periodontitis [24]. 

To compare the potency of PMFs between nobiletin and tangeretin, we added 10 μg/mL of the PMF mixture, as well as nobiletin and tangeretin alone, to a coculture of POBs and BMC for IL-1-induced osteoclast differentiation. At this sub-optimum dose, PMFs suppressed 84% of IL-1-indued osteoclast differentiation, whereas nobiletin and tangeretin showed 62% and 18% suppression, respectively. Therefore, the potency of PMFs was deemed to be higher than that of nobiletin and tangeretin. Since our PMFs mixture contained nobiletin, tangeretin, HMF, and TMF, it is possible that these components exhibit co-operative effects for the suppression of osteoclastogenesis. Further studies are needed to determine the structure-activity correlation and molecular mechanism underlying the synergetic effects among the respective component of the PMF mixture against bone metabolism.

In the present study, the PMF mixture significantly suppressed the IL-1-induced osteoclast differentiation and bone-resorbing activity in vitro and restored bone loss in OVX mice in vivo. Murakami et al. [30] demonstrated that the treatment of nobiletin prevented bone loss in OVX mice and also attenuated type II collagen-induced arthritis in mice. In the present study, the oral administration of PMFs (5 mg/day/mouse) significantly restored the loss of trabecular bone due to estrogen deficiency in OVX mice (Figure 2 and Figure 3). In sham mice, the administration of PMFs enhanced the femoral BMD as measured by DEXA in the central area consisting only of cortical bone (Figure 2D), but the trabecular bone volume as measured by μCT was not affected by PMFs (Figure 3B). Bone turnover and bone formation rate are different between trabecular bone and cortical bone. Therefore, PMFs may stimulate bone formation in cortical bone, but further studies are needed to confirm this possibility. In our previous study, the intraperitoneal injection of nobiletin (2 mg/day/ mouse) restored femoral bone loss in OVX mice [23], but the oral administration of PMFs (5 mg/day/mouse) was found to be sufficient to recover bone mass in OVX mice in the present study. Oral administration generally requires more than 10-fold the dose required with intraperitoneal treatment. Therefore, PMFs may exhibit more potent effects on bone mass than nobiletin in OVX mice. 

We previously reported that LPS-TLR4 signaling induced PGE_2_-mediated inflammatory bone resorption [6]. LPS, a bacterial component, is a known pathogen of periodontitis and has also been identified as a ligand for TLR4. Choi et al. [31] reported that nobiletin inhibits LPS-induced COX-2 expression and ROS production in Raw 264.7 cells, a mouse osteoclast precursor cell line, through the attenuation of DNA-binding activity of NFκB. Shu et al. [32] found that tangeretin suppresses the LPS-induced production of inflammatory molecules, including IL-6, tumor necrosis factor (TNF)-α, and PGE_2_, via the modulation of NFκB activation in microglial cells. We previously reported that nobiletin, tangeretin, and HMF inhibit LPS-induced PGE production via osteoblasts and bone resorption [24,25]. In the present study, PMFs suppressed LPS-stimulated osteoclast differentiation in the coculture of POB and BMCs and inhibited the bone-resorbing activity in calvarial organ cultures (Figure 4). PMFs also restored alveolar bone resorption in organ cultures of mandibular alveolar bone in an experimental model of periodontitis (Figure 4C). Given the increasing number of aged patients, the prevalence of bone resorption disorders is expected to increase. PMFs may be potential candidate compounds for the prevention and treatment of bone diseases, including osteoporosis and periodontitis. Since PMFs did not affect uterine weight in OVX mice, oral administration of PMFs may be useful for the treatment of postmenopausal osteoporosis in women without side effects in uterus. PMFs is natural compound derived from citrus and generally safety, but further studies are needed to define a possible side effect in human. In addition, local application of PMFs in periodontal tissues may also be useful for the prevention of periodontitis.

## 4. Materials and Methods

### 4.1. Animals and Reagents

Newborn and 5- and 6-week-old mice of the ddY strain were obtained from Japan SLC Inc. (Shizuoka, Japan). All procedures were performed in accordance with the institutional guidelines for animal research. PMFs were provided as an original mixture of nobiletin, tangeretin, HMF, and TMF (35.7%, 11.0%, 2.4%, and 38.8%, respectively) by Yasuhara Chemical Co., Ltd. (Hiroshima, Japan). IL-1 was purchased from R&D Systems (Minneapolis, MN, USA). The identification of respective PMF was justified by HPLC. LPS from Escherichia coli was obtained from Sigma-Aldrich Co. LLC. (St. Louis, MO, USA).

### 4.2. Isolation of Primary Mouse Osteoblastic Cells

Primary osteoblastic cells (POBs) were collected from newborn mouse calvariae after five routine sequential digestions with 0.1% collagenase (Roche Diagnostics GmbH, Mannheim, Germany) and 0.2% dispase (Roche Applied Science, Mannheim, Germany). POBs were cultured in α-modified MEM (αMEM) supplemented with 10% fetal bovine serum (FBS) at 37 °C under 5% CO_2_ in air.

### 4.3. Co-Cultures of Mouse Bone Marrow Cells and Osteoblasts

Bone marrow cells (BMCs) were isolated from tibia in 6-week-old mice. BMCs (2 × 10^6^ cells) and POBs (1 × 10^4^ cells) were cocultured with IL-1 (2 ng/mL) or LPS (1 ng/mL) in the presence of PMFs (15, 30 μg/mL) in αMEM containing 10% FBS for 7 days. The cells were fixed with 10% formaldehyde and stained for tartrate-resistant acid phosphatase (TRAP). TRAP-positive multinucleated cells per well were counted as osteoclasts.

### 4.4. Organ Cultures of Mouse Calvariae

Newborn mouse calvariae precultured for 24 h in BGJb medium with 0.1% bovine serum albumin (BSA) at 37 °C under 5% CO_2_ in the air. Calvariae were treated with IL-1 (4 ng/mL) or LPS (1 μg/mL) in the presence or absence of PMFs (15, 30 μg/mL) and cultured for 5 days. The bone-resorbing activity was elucidated by measuring the concentration of calcium in the conditioned medium using the o-cresolphthalein complexone (OCPC) method, as reported previously [8].

### 4.5. Oral Administration of PMFs in OVX Mice

Five-week-old female mice were either sham-operated or ovariectomized (OVX). PMFs was diluted with sesame oil, and Gum arabic was added to the PMF/sesame oil and mixed with water. The PMF solution (5 mg; 250 μL) was orally administered to mice daily using a sonde for 4 weeks. After 4 weeks, the femurs were collected, and the bone mass was measured by dual X-ray absorptiometry (DXA) (model DCS-600R; Aloka, Tokyo, Japan) and micro-computed tomography (μCT) (inspeXio SMX-90CT; Shimadzu, Kyoto, Japan). In DXA, the scanned area of total BMD was divided equally into three regions: proximal, central, and distal femur, to assess the regional differences in femoral BMD. 

### 4.6. Organ Cultures of Mouse Mandibular Alveolar Bone

Mouse mandibular bones without teeth were collected from 5-week-old mice and precultured for 24 h in BGJb medium with 0.1% BSA at 37 °C under 5% CO_2_ in the air. Mandibular bones were treated with LPS (1 μg/mL) in the presence or absence of PMFs (15 μg/mL) and cultured for 5 days. The bone-resorbing activity was elucidated by measuring the increased medium calcium using OCPC methods.

### 4.7. Statistical Analyses

Data are presented as the means ± standard error of the mean (SEM). The significance of differences was analyzed using Student’s *t*-test.
